# Dibromido{2-[1-(cyclo­propyl­imino)­eth­yl]pyridine}­zinc(II)

**DOI:** 10.1107/S1600536810025201

**Published:** 2010-07-03

**Authors:** Si-Yu Yue, Jiu-Fu Lu

**Affiliations:** a School of Chemistry and Environmental Science, Shaanxi University of Technology, Hanzhong 723000, People’s Republic of China

## Abstract

In the title compound, [ZnBr_2_(C_10_H_12_N_2_)], the Zn^2+^ ion is coordinated by the *N*,*N*′-bidentate Schiff base ligand and two bromode ions in a distorted tetra­hedral arrangement. The dihedral angle between the pyridine and the cyclo­propyl rings is 95.4 (8)°.

## Related literature

For background to Schiff bases as chelating ligands, see: Hamaker *et al.* (2010 ▶); Wang *et al.* (2010 ▶); Mirkhani *et al.* (2010 ▶); Liu & Yang (2009 ▶). For similar zinc complexes, see: Zakrzewski & Lingafelter (1970 ▶); Gourbatsis *et al.* (1999 ▶); Merino *et al.* (2001 ▶); Majumder *et al.* (2006 ▶).
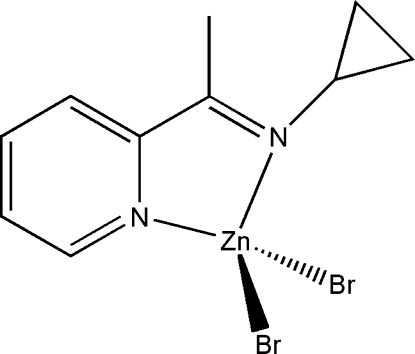


## Experimental

### 

#### Crystal data

[ZnBr_2_(C_10_H_12_N_2_)]
*M* *_r_* = 385.41
Monoclinic, 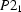

*a* = 7.029 (3) Å
*b* = 14.090 (3) Å
*c* = 7.037 (2) Å
β = 111.820 (3)°
*V* = 647.0 (4) Å^3^
*Z* = 2
Mo *K*α radiation
μ = 8.04 mm^−1^
*T* = 298 K
0.23 × 0.23 × 0.21 mm

#### Data collection

Bruker APEXII CCD diffractometer
Absorption correction: multi-scan (*SADABS*; Sheldrick, 2004 ▶) *T*_min_ = 0.259, *T*_max_ = 0.283
4060 measured reflections
2408 independent reflections
1708 reflections with *I* > 2σ(*I*)
*R*_int_ = 0.104

#### Refinement

*R*[*F* ^2^ > 2σ(*F* ^2^)] = 0.063
*wR*(*F* ^2^) = 0.165
*S* = 0.95
2408 reflections
137 parameters
1 restraint
H-atom parameters constrained
Δρ_max_ = 0.96 e Å^−3^
Δρ_min_ = −1.09 e Å^−3^
Absolute structure: Flack (1983 ▶), 957 Friedel pairs
Flack parameter: −0.05 (3)

### 

Data collection: *APEX2* (Bruker, 2004 ▶); cell refinement: *SAINT* (Bruker, 2004 ▶); data reduction: *SAINT*; program(s) used to solve structure: *SHELXS97* (Sheldrick, 2008 ▶); program(s) used to refine structure: *SHELXL97* (Sheldrick, 2008 ▶); molecular graphics: *SHELXTL* (Sheldrick, 2008 ▶); software used to prepare material for publication: *SHELXTL*.

## Supplementary Material

Crystal structure: contains datablocks global, I. DOI: 10.1107/S1600536810025201/hb5526sup1.cif

Structure factors: contains datablocks I. DOI: 10.1107/S1600536810025201/hb5526Isup2.hkl

Additional supplementary materials:  crystallographic information; 3D view; checkCIF report

## Figures and Tables

**Table 1:** Selected bond lengths (Å)

Zn1—N1	2.041 (9)
Zn1—N2	2.073 (10)
Zn1—Br1	2.3488 (18)
Zn1—Br2	2.3616 (19)

**Fig. 1.**
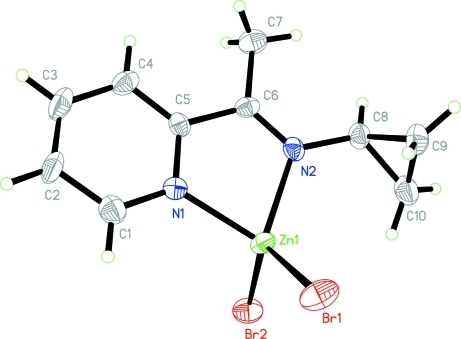
The molecular structure of the title complex, showing 30% probability displacement ellipsoids.

## supplementary crystallographic information

### Comment

Schiff bases have often been used as chelating ligands in coordination chemistry (Hamaker *et al.*, 2010; Wang *et al.*, 2010; Mirkhani *et al.*, 2010; Liu & Yang, 2009). We report here the crystal structure of the title new zinc complex with the chelating Schiff base ligand cyclopropyl-(1-pyridin-2-ylethylidene)amine.

The Zn atom in the complex is four-coordinated by one pyridine N and one imine N atoms of the Schiff base ligand, and by two bromide atoms, forming a tetrahedral geometry (Fig. 1). The dihedral angle between the pyridine and the cyclopropyl rings is 95.4 (8)°. The bond lengths (Table 1) related to the Zn atom are comparable to those observed in similar zinc complexes (Zakrzewski & Lingafelter, 1970; Gourbatsis *et al.*, 1999; Merino *et al.*, 2001; Majumder *et al.*, 2006).

### Experimental

2-Acetylpyridine (0.1 mmol, 12.1 mg) and cyclopropylamine (0.1 mmol, 5.7 mg) were mixed and stirred in methanol (10 ml) for 30 min. Then a methanol solution (5 ml) of zinc bromide (0.1 mmol, 22.5 mg) was added to the mixture. The final mixture was stirred for another 30 min to give a colourless solution. Colourless blocks of (I) were obtained by slow evaporation of the solution at room temperature.

### Refinement

H atoms were positioned geometrically (C—H = 0.93–0.98 Å) and refined using a riding model, with with *U*_iso_(H) = 1.2 or 1.5*U*_eq_(C). A rotating group model was used for the methyl group.

### Crystal data

**Table:**

[ZnBr_2_(C_10_H_12_N_2_)]	*F*(000) = 372
*M**_r_* = 385.41	*D*_x_ = 1.978 Mg m^−^^3^
Monoclinic, *P*2_1_	Mo *K*α radiation, λ = 0.71073 Å
*a* = 7.029 (3) Å	Cell parameters from 1405 reflections
*b* = 14.090 (3) Å	θ = 2.8–25.0°
*c* = 7.037 (2) Å	µ = 8.04 mm^−^^1^
β = 111.820 (3)°	*T* = 298 K
*V* = 647.0 (4) Å^3^	Block, colourless
*Z* = 2	0.23 × 0.23 × 0.21 mm

### Data collection

**Table:**

Bruker APEXII CCD diffractometer	2408 independent reflections
Radiation source: fine-focus sealed tube	1708 reflections with *I* > 2σ(*I*)
graphite	*R*_int_ = 0.104
ω scans	θ_max_ = 27.0°, θ_min_ = 2.9°
Absorption correction: multi-scan (*SADABS*; Sheldrick, 2004)	*h* = −8→8
*T*_min_ = 0.259, *T*_max_ = 0.283	*k* = −18→17
4060 measured reflections	*l* = −8→8

### Refinement

**Table:**

Refinement on *F*^2^	Secondary atom site location: difference Fourier map
Least-squares matrix: full	Hydrogen site location: inferred from neighbouring sites
*R*[*F*^2^ > 2σ(*F*^2^)] = 0.063	H-atom parameters constrained
*wR*(*F*^2^) = 0.165	*w* = 1/[σ^2^(*F*_o_^2^) + (0.0966*P*)^2^] where *P* = (*F*_o_^2^ + 2*F*_c_^2^)/3
*S* = 0.95	(Δ/σ)_max_ < 0.001
2408 reflections	Δρ_max_ = 0.96 e Å^−^^3^
137 parameters	Δρ_min_ = −1.09 e Å^−^^3^
1 restraint	Absolute structure: Flack (1983), 957 Friedel pairs
Primary atom site location: structure-invariant direct methods	Flack parameter: −0.05 (3)

### Special details

**Table:**

Geometry. All esds (except the esd in the dihedral angle between two l.s. planes) are estimated using the full covariance matrix. The cell esds are taken into account individually in the estimation of esds in distances, angles and torsion angles; correlations between esds in cell parameters are only used when they are defined by crystal symmetry. An approximate (isotropic) treatment of cell esds is used for estimating esds involving l.s. planes.
Refinement. Refinement of F^2^ against ALL reflections. The weighted R-factor wR and goodness of fit S are based on F^2^, conventional R-factors R are based on F, with F set to zero for negative F^2^. The threshold expression of F^2^ > 2sigma(F^2^) is used only for calculating R-factors(gt) etc. and is not relevant to the choice of reflections for refinement. R-factors based on F^2^ are statistically about twice as large as those based on F, and R- factors based on ALL data will be even larger.

### Fractional atomic coordinates and isotropic or equivalent isotropic displacement parameters (Å^2^)

**Table:**

	*x*	*y*	*z*	*U*_iso_*/*U*_eq_	
Zn1	0.01583 (18)	0.10050 (8)	0.5412 (2)	0.0380 (3)	
Br1	−0.0650 (2)	−0.05302 (9)	0.6175 (2)	0.0601 (4)	
Br2	0.0576 (2)	0.12235 (9)	0.2262 (2)	0.0559 (4)	
N1	−0.1663 (14)	0.2047 (7)	0.5832 (15)	0.040 (2)	
N2	0.2313 (14)	0.1748 (7)	0.7783 (14)	0.036 (2)	
C1	−0.3710 (18)	0.2147 (10)	0.491 (2)	0.050 (3)	
H1	−0.4447	0.1674	0.4022	0.060*	
C2	−0.477 (2)	0.2918 (10)	0.524 (2)	0.053 (3)	
H2	−0.6184	0.2965	0.4582	0.064*	
C3	−0.369 (2)	0.3597 (10)	0.652 (2)	0.057 (4)	
H3	−0.4351	0.4134	0.6742	0.069*	
C4	−0.158 (2)	0.3503 (9)	0.753 (2)	0.047 (3)	
H4	−0.0833	0.3966	0.8450	0.056*	
C5	−0.0622 (16)	0.2727 (8)	0.7166 (16)	0.034 (2)	
C6	0.1628 (17)	0.2531 (8)	0.8195 (16)	0.036 (2)	
C7	0.288 (2)	0.3276 (9)	0.968 (2)	0.058 (4)	
H7A	0.2300	0.3390	1.0702	0.087*	
H7B	0.2870	0.3854	0.8960	0.087*	
H7C	0.4265	0.3057	1.0337	0.087*	
C8	0.4414 (17)	0.1441 (9)	0.8788 (19)	0.044 (3)	
H8	0.5438	0.1939	0.9385	0.053*	
C9	0.471 (2)	0.0526 (10)	0.995 (2)	0.051 (3)	
H9A	0.3501	0.0188	0.9917	0.061*	
H9B	0.5889	0.0474	1.1220	0.061*	
C10	0.511 (2)	0.0589 (11)	0.799 (2)	0.058 (4)	
H10A	0.6518	0.0572	0.8084	0.070*	
H10B	0.4131	0.0285	0.6781	0.070*	

### Atomic displacement parameters (Å^2^)

**Table:**

	*U*^11^	*U*^22^	*U*^33^	*U*^12^	*U*^13^	*U*^23^
Zn1	0.0393 (6)	0.0335 (7)	0.0441 (7)	−0.0027 (6)	0.0189 (5)	−0.0084 (6)
Br1	0.0749 (9)	0.0420 (7)	0.0784 (10)	−0.0192 (7)	0.0460 (8)	−0.0117 (7)
Br2	0.0672 (8)	0.0607 (9)	0.0464 (7)	−0.0071 (6)	0.0287 (7)	−0.0038 (6)
N1	0.039 (5)	0.039 (6)	0.039 (5)	0.000 (4)	0.010 (4)	−0.005 (4)
N2	0.040 (5)	0.037 (5)	0.029 (5)	0.004 (4)	0.011 (4)	0.009 (4)
C1	0.043 (7)	0.061 (8)	0.045 (7)	0.001 (6)	0.016 (6)	0.001 (6)
C2	0.056 (8)	0.056 (8)	0.056 (8)	0.029 (7)	0.030 (7)	0.009 (7)
C3	0.069 (9)	0.045 (8)	0.066 (9)	0.028 (7)	0.035 (8)	0.013 (7)
C4	0.066 (8)	0.034 (6)	0.045 (7)	0.006 (6)	0.026 (7)	0.001 (5)
C5	0.035 (6)	0.038 (6)	0.028 (5)	0.003 (5)	0.009 (5)	0.009 (5)
C6	0.051 (7)	0.031 (6)	0.030 (6)	−0.002 (5)	0.020 (5)	0.001 (4)
C7	0.065 (9)	0.041 (8)	0.061 (9)	−0.007 (6)	0.016 (8)	−0.014 (6)
C8	0.032 (6)	0.043 (7)	0.052 (7)	0.002 (5)	0.009 (5)	0.001 (6)
C9	0.052 (8)	0.047 (7)	0.054 (7)	0.012 (6)	0.019 (7)	0.015 (6)
C10	0.046 (7)	0.079 (10)	0.047 (7)	0.018 (7)	0.015 (6)	0.007 (7)

### Geometric parameters (Å, °)

**Table:**

Zn1—N1	2.041 (9)	C4—H4	0.9300
Zn1—N2	2.073 (10)	C5—C6	1.500 (15)
Zn1—Br1	2.3488 (18)	C6—C7	1.514 (16)
Zn1—Br2	2.3616 (19)	C7—H7A	0.9600
N1—C1	1.348 (15)	C7—H7B	0.9600
N1—C5	1.350 (15)	C7—H7C	0.9600
N2—C6	1.279 (15)	C8—C10	1.48 (2)
N2—C8	1.446 (14)	C8—C9	1.498 (18)
C1—C2	1.383 (18)	C8—H8	0.9800
C1—H1	0.9300	C9—C10	1.506 (19)
C2—C3	1.34 (2)	C9—H9A	0.9700
C2—H2	0.9300	C9—H9B	0.9700
C3—C4	1.392 (19)	C10—H10A	0.9700
C3—H3	0.9300	C10—H10B	0.9700
C4—C5	1.358 (17)		
			
N1—Zn1—N2	80.2 (4)	N2—C6—C5	117.9 (10)
N1—Zn1—Br1	114.3 (3)	N2—C6—C7	125.7 (11)
N2—Zn1—Br1	116.6 (3)	C5—C6—C7	116.4 (10)
N1—Zn1—Br2	110.2 (3)	C6—C7—H7A	109.5
N2—Zn1—Br2	112.4 (3)	C6—C7—H7B	109.5
Br1—Zn1—Br2	117.36 (7)	H7A—C7—H7B	109.5
C1—N1—C5	117.7 (10)	C6—C7—H7C	109.5
C1—N1—Zn1	128.4 (8)	H7A—C7—H7C	109.5
C5—N1—Zn1	113.8 (7)	H7B—C7—H7C	109.5
C6—N2—C8	123.3 (11)	N2—C8—C10	118.4 (11)
C6—N2—Zn1	113.2 (7)	N2—C8—C9	115.8 (10)
C8—N2—Zn1	123.4 (8)	C10—C8—C9	60.7 (9)
N1—C1—C2	123.2 (13)	N2—C8—H8	116.7
N1—C1—H1	118.4	C10—C8—H8	116.7
C2—C1—H1	118.4	C9—C8—H8	116.7
C3—C2—C1	117.8 (12)	C8—C9—C10	59.1 (9)
C3—C2—H2	121.1	C8—C9—H9A	117.9
C1—C2—H2	121.1	C10—C9—H9A	117.9
C2—C3—C4	120.3 (12)	C8—C9—H9B	117.9
C2—C3—H3	119.8	C10—C9—H9B	117.9
C4—C3—H3	119.8	H9A—C9—H9B	115.0
C5—C4—C3	119.3 (12)	C8—C10—C9	60.2 (8)
C5—C4—H4	120.4	C8—C10—H10A	117.8
C3—C4—H4	120.4	C9—C10—H10A	117.8
N1—C5—C4	121.6 (10)	C8—C10—H10B	117.8
N1—C5—C6	114.0 (10)	C9—C10—H10B	117.8
C4—C5—C6	124.3 (11)	H10A—C10—H10B	114.9
